# I Can't Take My Eyes Off of You: Attentional Allocation to Infant, Child, Adolescent and Adult Faces in Mothers and Non-Mothers

**DOI:** 10.1371/journal.pone.0109362

**Published:** 2014-10-29

**Authors:** Chloe Thompson-Booth, Essi Viding, Linda C. Mayes, Helena J. V. Rutherford, Sara Hodsoll, Eamon McCrory

**Affiliations:** 1 Division of Psychology and Language Sciences, University College London, London, United Kingdom; 2 Yale Child Study Center, School of Medicine, Yale University, New Haven, Connecticut, United States of America; University of Portsmouth, United Kingdom

## Abstract

It has been reported previously that infant faces elicit enhanced attentional allocation compared to adult faces in adult women, particularly when these faces are emotional and when the participants are mothers, as compared to non-mothers [1]. However, it remains unclear whether this increased salience of infant faces as compared to adult faces extends to children older than infant age, or whether infant faces have a unique capacity to elicit preferential attentional allocation compared to juvenile or adult faces. Therefore, this study investigated attentional allocation to a variety of different aged faces (infants, pre-adolescent children, adolescents, and adults) in 84 adult women, 39 of whom were mothers. Consistent with previous findings, infant faces were found to elicit greater attentional engagement compared to pre-adolescent, adolescent, or adult faces, particularly when the infants displayed distress; again, this effect was more pronounced in mothers compared to non-mothers. Pre-adolescent child faces were also found to elicit greater attentional engagement compared to adolescent and adult faces, but only when they displayed distress. No preferential attentional allocation was observed for adolescent compared to adult faces. These findings indicate that cues potentially signalling vulnerability, specifically age and sad affect, interact to engage attention. They point to a potentially important mechanism, which helps facilitate caregiving behaviour.

## Introduction

Faces are a special class of stimuli that preferentially engage our attention, providing valuable information essential for successful social interaction and survival [2]–[4]. Glimpsing a face, even momentarily, provides us with a wealth of information about an individual's identity, age, gender, ethnic background and emotional state [5]. However, as faces are common stimuli in most human environments there is a need to selectively deploy attentional resources to those faces that signal potentially important information [6], [7]. For example, facial expression of emotion and the age of a face are likely to provide information to inform appropriate social interactions and responses [2], [7]–[11].

The presence of emotional content is perhaps the most robust feature known to influence attention to faces [7], [12]; it is well established that attention is greater for emotional than neutral faces [13], [14]. For example, Hodsoll and colleagues (2011) demonstrated attentional capture by emotional distractor faces (fearful, angry, or happy) in a search task in which emotional expression was entirely irrelevant. Other studies have demonstrated that faces expressing positive and negative emotion differ in the relative effectiveness with which they capture attention as compared to neutral faces; faces expressing negative emotion guide focal attention more effectively than do faces expressing positive emotion [15], [16]. It has also been found that threatening faces are detected more quickly than friendly faces among neutral, emotional or sad distractors, and that fearful and angry faces elicit similar biases in visuospatial orienting [17], [18]. Response to facial threat is in fact often rapid and even unconscious in manner [12], consistent with the view that it is evolutionarily adaptive to preferentially attend and respond to threat-related stimuli which may signal that an individual is in danger [19], [20].

Taken together, these studies suggest that emotional faces influence the allocation of attention, and that these effects are most marked for faces that signal we (and/or others) may be at risk of harm. However, the majority of studies investigating the effects of facial emotion on attention have used only adult stimuli.

Faces also provide a rich source of information about a person's age, which can also influence how we attend to them [21]–[24]. Particularly robust effects of face age relate to the attentional capture effects of baby schema [21], [22], typically characterized by a large round face, high and protruding forehead, large eyes, small mouth and nose [11], [25]. These baby schema features are observed in the young of many species, and it has been argued that they function as an evolutionary convergence phenomenon that allow the recognition of young age and heightened need for care from an infant's parents but also from other adults and even different species [11], [26]. Arguably these specific perceptual features, which delineate young age, also indicate heightened vulnerability and need for care [11]. Lorenz proposed that this infant-specific configuration (“Kindchenschema”) acts as an innate releasing mechanism for caretaking behaviour and affective orientation towards infants, with the evolutionary function of enhancing offspring survival [27]–[29].

Consistent with Lorenz's proposal, it has been found that both children and adults prefer pictures of younger infants and infants exhibiting higher levels of Kindchenschema features [30]–[32]. It has also been noted in primates that the loss of infantile characteristics as offspring age typically coincides with a reduction in parental responses [33]. Furthermore, neuroimaging research has begun to show that infant faces might be processed differently to adult faces. Compared to adult faces, infant faces elicit enhanced activation in a distributed network implicated in face perception, reward processing and attentional processing [34], [35]. Parametrically manipulating baby schema content to make them ‘cuter’ is associated with greater activation of the nucleus accumbens [36], consistent with the view that baby schema represent a rewarding sensory stimulus that may motivate caretaking behaviour.

Moreover, recent studies have found that adults show enhanced attentional allocation to infant compared to adult faces [1], [21], [37]. These studies have shown attentional capture to infant faces over adult faces in a non-parent sample [21] and delayed disengagement from infant faces as compared to adult faces, particularly if these faces display distress, in a group of pregnant women [37]. A recent study by Thompson-Booth and colleagues (2014) investigated attentional responding to infant and adult faces displaying different facial expressions in a group of mothers and non-mothers using a visual search paradigm. Participants were asked to respond to the orientation of a target face (defined by eye colour), which was displayed with two non-target faces. Importantly, emotional expression and face identity were not relevant to the task, so by measuring response times in different conditions (emotion vs. neutral; adult faces vs. infant faces) it was possible to discern whether non-target features of the scene (faces or emotion) were engaging attention and interfering with task performance. It was found that response times were slowed in the presence of infant faces as compared to adult faces, particularly if they displayed an emotional expression. Furthermore, mothers appeared to show particularly slowed responses to infant faces. Slowed responses were interpreted as indicative of enhanced allocation of attention towards processing face age and emotion, rather than responding to the task-specific features of the scene. Taken together, these studies further support the contention that infant faces are particularly salient.

Preferential allocation of attention to infant faces compared to adult faces makes evolutionary sense as it may help ensure survival of those who are entirely dependent on others for food, shelter and comfort. However, this evolutionary mechanism may be further sensitized in parents, who have consistent caregiving responsibilities. Both behavioural and neuroimaging studies suggest that mothers of infants process infant cues differently to non-mothers [1], [38]. Furthermore, mothers and pregnant women appear to find emotional infant faces particularly engaging [1], [37]. These findings suggest that parenthood may be associated with a greater empathic response or increased arousal to infant faces [39].

Despite this growing body of research demonstrating that infant faces may be a particularly special class of social stimuli, there remains surprisingly scant empirical evidence regarding attentional processing of children's faces outside of infancy. It is not clear whether increased salience of infant faces as compared to adult faces extends to children older than infant age, which would suggest that young faces in general have privileged access to attention, or whether infancy alone demands preferential attentional allocation as compared to a variety of other aged faces. Outside of infancy, children continue to remain relatively dependent on adult care to meet their emotional and physical needs, however there is a reduction over time in the adult nurturance that they require, and significant cross-cultural differences in this regard [40], [41]. Furthermore, as faces age, the degree of baby schema they express lessens, with infants having the strongest baby schema characteristics before the age of 1 year [42]. The findings of a recent electrophysiological study showed a larger face-specific neural response in women to infant than to child and adult faces. They also found that a neural response associated with brain areas involved in face and reward processing was affected by face age, with larger amplitudes to infant faces than to child faces, and larger amplitudes to child faces than to adult faces [43]. These findings are in line with the notion that we exhibit a preferential response to infant faces, but are at least suggestive that children's faces may also be processed preferentially compared to adult faces.

Given the absence of research investigating attentional processing of child faces beyond infancy, the present study aimed to replicate and extend previous research by investigating whether adults differentially attend to faces from different age groups using a previously established visual search task [1], [14]. Specifically we wished to establish whether the observed pattern of enhanced attention to infant faces extends to the faces of older children and adolescents expressing neutral or sad affect, as compared to adult faces. Based on previous findings, we predicted that visual search task RTs would be slowed in response to the faces of both infants and children [1], [43], with infant effects enhanced in parents who had young children themselves as compared to a non-parent group [1]. No specific predictions were made in relation to differential processing of child faces by parents and non-parents given the lack of previous research comparing these groups on a similar task. We also predicted that facial expressions of sadness would enhance attentional allocation to faces as compared to neutral affect, particularly for infant and child faces, based on previous studies using infant stimuli which found that emotional expressions demand more attention than a neutral expression [1], [37]. No differences in attentional processing were predicted in relation to adolescent faces, as this stage was hypothesized to reflect a relatively autonomous developmental period [41].

## Method

### Ethics statement

**Figure 1:**
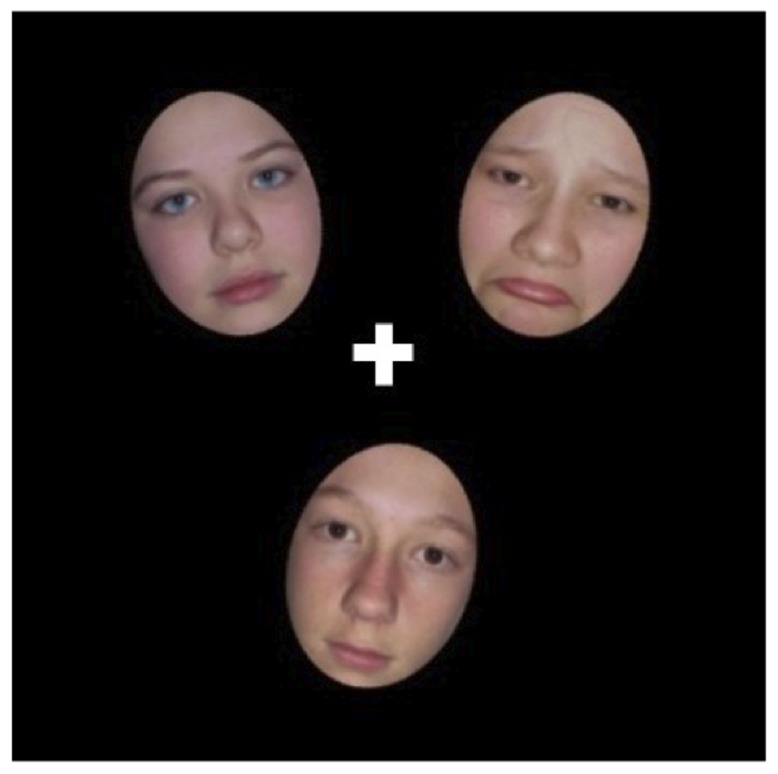
Examples of stimuli used in task (not to scale). This example shows adolescent stimuli with an emotional non-target face present.

The study was granted ethical approval from University College London ethics board (approval number 2407/001). Participants gave written informed consent to participate in the study. The individuals who appear in Figure 1 in this manuscript have given written informed consent (as outlined in PLOS consent form) to publish this photograph.

### Participants

Eighty-four women, 39 first-time mothers and 45 non-mothers, were recruited for the study from the Psychology Department Subject Pool and local community. The women were aged between 23 and 39 years old (mothers: *M* = 29.95 years, *SD* = 4.90; non-mothers: *M* = 28.22 years, *SD* = 4.25; *t*(82) = −1.72; *p* = .09). All participants were white and were matched for age, IQ, household income, and number of years in education (see Table 1). All participants reported normal or corrected-to-normal vision and were right handed. All of the mothers had a singleton pregnancy and their children were aged between 2 and 30 months (*M* = 15.79 months, *SD* = 9.74).

**Table 1:** Participant demographics.

	Mothers (N = 39)		Non-Mothers (N = 45)		
	Mean (SD)	Range	Mean (SD)	Range	*P* value
Age	29.95 (4.9)	23–39	28.22 (4.26)	23–37	.10
WASI 2-subtest estimated FSIQ*	112.28 (7.0)	101–133	114.32 (7.8)	99–135	.11
Years in education	16.67 (2.8)	12–22	17.44 (1.6)	15–23	.22
Household income	*n*	%	*n*	%	
£0–£15,000	8	20.51	13	28.89	.40
£15,000–£30,000	8	20.51	12	26.67	
£30,000–£50, 000	10	25.64	12	26.67	
£50,000+	13	33.33	8	17.78	

* WASI data was missing from one non-mother.

### Apparatus

The visual search task was conducted using a Sony Vaio Windows 7 PC laptop with a 2.4-GHz Intel Core Duo processor and a 13″ wide screen monitor (60 Hz, 1366×768 resolution). Stimuli were presented and RTs recorded using Psytools software (Delosis Limited).

### Stimuli

An established visual search task [1], [14] was employed and adapted for the current study. Participants were asked to select one “odd” face out among three faces according to eye colour and indicate with a button press if the target face was tilted right or left. This task has been previously shown to enable a reliable indexing of enhanced attention to facial affect and infant status [1], [14]. It is hypothesised that these cues are sufficiently salient to involuntarily engage attention, slowing reaction time in the visual search task.

The version of the task employed in this study contained colour images of white male and female faces of different ages; infants (N = 4; aged 6–12 months), children (N = 4; aged 4–7 years old), adolescents (N = 4; aged 13–16 years old), and adults (N = 4; aged 30–45 years old). Images were provided by Baylor College of Medicine [44], the MacBrain Face Stimulus Set [45], and from photographs taken by the authors. There were images of each identity showing neutral and sad facial expressions. In a preliminary study, 14 individuals who did not take part in the main study rated all images for age, valence, arousal, and vulnerability on a scale of 1–5 (*See supplementary information S1*).

All of the images were edited using Paint.net software so that each identity displayed blue eyes on some trials (when target) and brown eyes on other trials (when non-target). Eye-size (measured in pixels) was matched across stimuli. Each of the faces subtended 26 mm (vertically) by 21 mm (horizontally). On any one trial three faces from the same age group were presented on a black background in a virtual triangle with the centre of each image placed at 24 mm from a central fixation cross (see Figure 1). Viewing distance was 60 cm.

### Design

Trials were blocked by face age, with the order randomised across participants. Each block consisted 72 trials; within each block, two thirds of the trials (48 trials) were neutral conditions in which no emotional faces were present. On the other third (24 trials) a sad facial expression was present on one of the faces; in half of these trials (12 trials) sadness was present on one of the brown-eyed non-target faces and in the other half of the trials sadness was present on the blue-eyed target face. Taking all the conditions together, a 4 (Face age: Infant, Pre-adolescent Child, Adolescent or Adult)×3 (Search condition: Emotional target, emotional non-target, and all neutral) repeated-measures design was employed, resulting in 12 experimental conditions. Within each block, the search condition (i.e. whether sad faces were absent, or whether sadness was present on the target face or one of the non-target face) was randomised across trials.

### Procedure

Participants were given instructions at the beginning the task, followed by practice sessions of 12 trials. Participants were instructed to search for a blue-eyed target face in a display with two brown-eyed non-target faces. Each of the three faces in the display were tilted either 15° to the left or 15° to the right (orientation was randomised). Participants were required to indicate when they had found the target face by responding to the orientation of the target face, pushing the left mouse key if the target face was tilted to the left and the right mouse key if tilted to the right. Participants were instructed to keep their left hand on the left key and their right hand on the right key in order to speed up responses. Participants were also asked to be as fast and accurate in their responses as possible. After a response the stimuli were removed from the screen and replaced with a central fixation cross. There was 500 ms between the onset of the fixation cross and the onset of the next stimuli. Stimuli remained on screen until a response was made, but a trial was aborted if no response was registered within 3000 ms. Auditory feedback (100 ms tone) was given if an incorrect response was made. After completing the attention task participants were assessed for general cognitive ability using the two-subtest form of the Wechsler Abbreviated Scale of Intelligence (Wechsler, 1999).

## Results

Anticipatory (<150 ms) responses (.01%) and incorrect responses (3.86% of total trials) were excluded from the reaction time (RT) analysis. Outliers (2.5 SDs from mean) were calculated for each participant's range of RTs and removed from analysis (2.48% of total trials), and mean correct RTs for each experimental condition were then calculated for analysis. Means and standard errors of reaction times are presented in Table 2. Correlation analyses were performed to assess whether participant age was associated with task performance. There was not a statistically significant correlation between age and RT for either mothers (*r* = .19, *p* = .24) or non-mothers (*r* = .11, *p* = .48) and therefore age was not included as a covariate in the analyses reported here.

**Table 2:** Descriptive Statistics for Reaction Time (ms) for all Trial Conditions for Mothers and Non-Mothers.

	Non-Mother (N = 45)						Mother (N = 39)					
	Neutral Search Condition		Sad Non-Target Search Condition		Sad Target Search Condition		Neutral Search Condition		Sad Non-Target Search Condition		Sad Target Search Condition	
	Mean	SE	Mean	SE	Mean	SE	Mean	SE	Mean	SE	Mean	SE
Infant faces	924.06	25.10	922.86	26.59	957.58	27.95	1083.75	26.96	1105.20	28.57	1164.69	30.03
Child faces	913.12	25.03	933.62	25.90	970.10	28.63	981.19	26.88	1009.34	27.82	1049.69	30.75
Adolescent faces	894.82	26.32	910.78	23.52	911.63	23.13	989.08	28.27	1002.84	25.27	1003.38	24.85
Adult faces	883.73	25.80	908.78	23.40	913.70	22.73	977.12	27.71	1006.44	25.13	1012.74	24.41

A 4 (face age: infant, child, adolescent, adult)×3 (search condition: sad target, sad non-target, and all neutral) repeated-measures ANOVA was conducted on the RT data, with parent status (mother or non-mother) entered as a between-subjects variable. Effect sizes are reported as partial eta squared (*η_p_^2^*) and Cohen's (*d*).

A main effect of face age was observed (Greenhouse-Geisser corrected *F*(2.6, 216.4) = 12.81, *p*<.001, *η_p_^2^* = .14). Pairwise comparisons were conducted with Bonferroni correction applied; reported *p*-values are adjusted values obtained from SPSS. These comparisons revealed that RTs were slower for infant face conditions than for adult face conditions (mean difference = 75.93 ms; *p*<.001, *d* = .67), slower for infant face conditions than for adolescent face conditions (mean difference = 74.26 ms; *p*<.001, *d* = .50), and slower for infant face conditions than for child face conditions (mean difference = 50.17 ms; *p*<.05, *d* = .29). There were no differences in RTs between adult and adolescent face conditions (mean difference = 1.67, *p* = 1.0, *d* = .02), or between adult and child face conditions (mean difference = 25.76, *p* = .36, *d* = .22). Finally, there were no differences in RTs between adolescent and child face conditions (mean difference  = , *p* = .55, *d* = .20).

There was a main effect of search condition (*F*(2, 164) = 29.31, *p*<.001, *η_p_^2^* = .26). Post-hoc pairwise comparisons (Bonferroni corrected *p*-values reported) indicated that participants' RTs to correct responses were slower in sad non-target conditions than in neutral conditions (mean difference = 19.12 ms, *p*<.01, *d* = .38), and slower in sad target conditions than in neutral conditions (mean difference = 42.08, *p*<.001, *d* = .76). Finally, RTs were slower in sad target conditions than in sad non-target conditions (mean difference = 22.95 ms, *p*<.001, *d* = .49).

**Figure 2:**
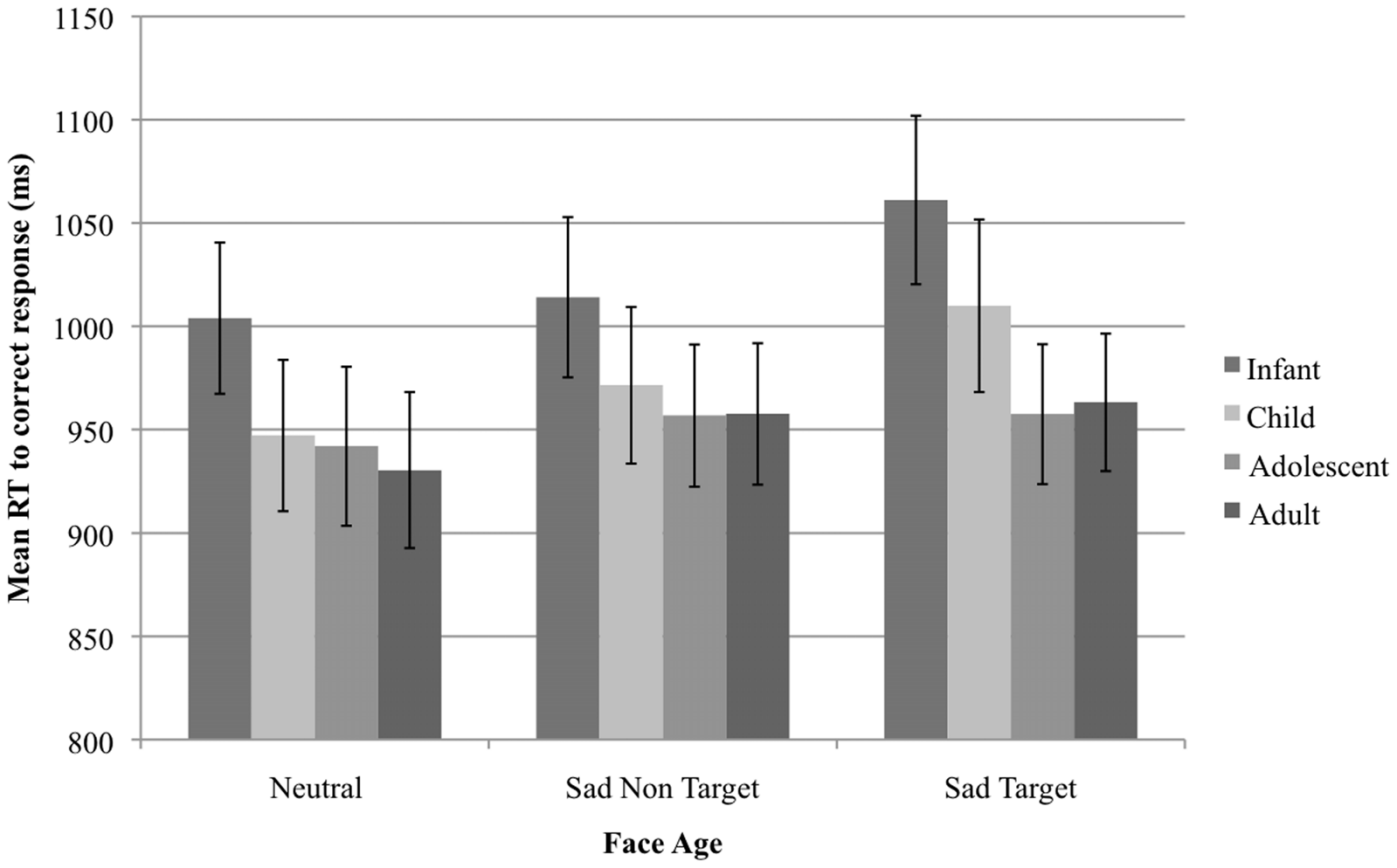
Mean RT for each experimental condition as a function of stimulus type. Error bars represent 95% confidence intervals.

There was also a face age by search condition interaction (Greenhouse-Geisser corrected *F*(5.1, 415.4) = 3.82, *p*<.001, *η_p_^2^* = .04). Post-hoc comparisons with Bonferroni corrections revealed that for neutral conditions, RTs were slower to infant faces than to adult (mean difference = 73.47 ms, *p*<.001, *d* = .56), adolescent (mean difference = 61.95 ms, *p*<.01, *d* = .35), and child faces (mean difference = 56.75 ms, *p*<.01, *d* = .33). However for sad non-target conditions, while RTs were slower to infant stimuli than adult (mean difference = 56.42 ms, *p*<.001, *d* = .43) and adolescent stimuli (mean difference = 57.22 ms, *p*<.05, *d* = .37), RTs were not slower to infant faces as compared to child faces (mean difference = 42.55, *p* = .10, *d* = .23). For sad target conditions, RTs were slower to infant faces than to adult (mean difference = 97.90 ms, *p*<.001, *d* = .67) and adolescent faces (mean difference = 103.62 ms, *p*<.001, *d* = .67), and approached statistical significance compared to child faces (mean difference = 51.23, *p* = .09, *d* = .24). Furthermore, RTs were slowed to child faces as compared to adolescent faces (mean difference = 52.39 ms, *p*<.05, *d* = .35), and adult faces (mean difference = 46.67 ms, *p*<.05, *d* = .33; *see* *Figure 2*). Therefore, while RTs were slower to infant faces than to adult and adolescent faces across all search conditions, RTs were only statistically slower to infant than to child in the neutral condition. Furthermore, RTs were slower to child faces than to adult and adolescent faces, but only in sad target conditions.

**Figure 3:**
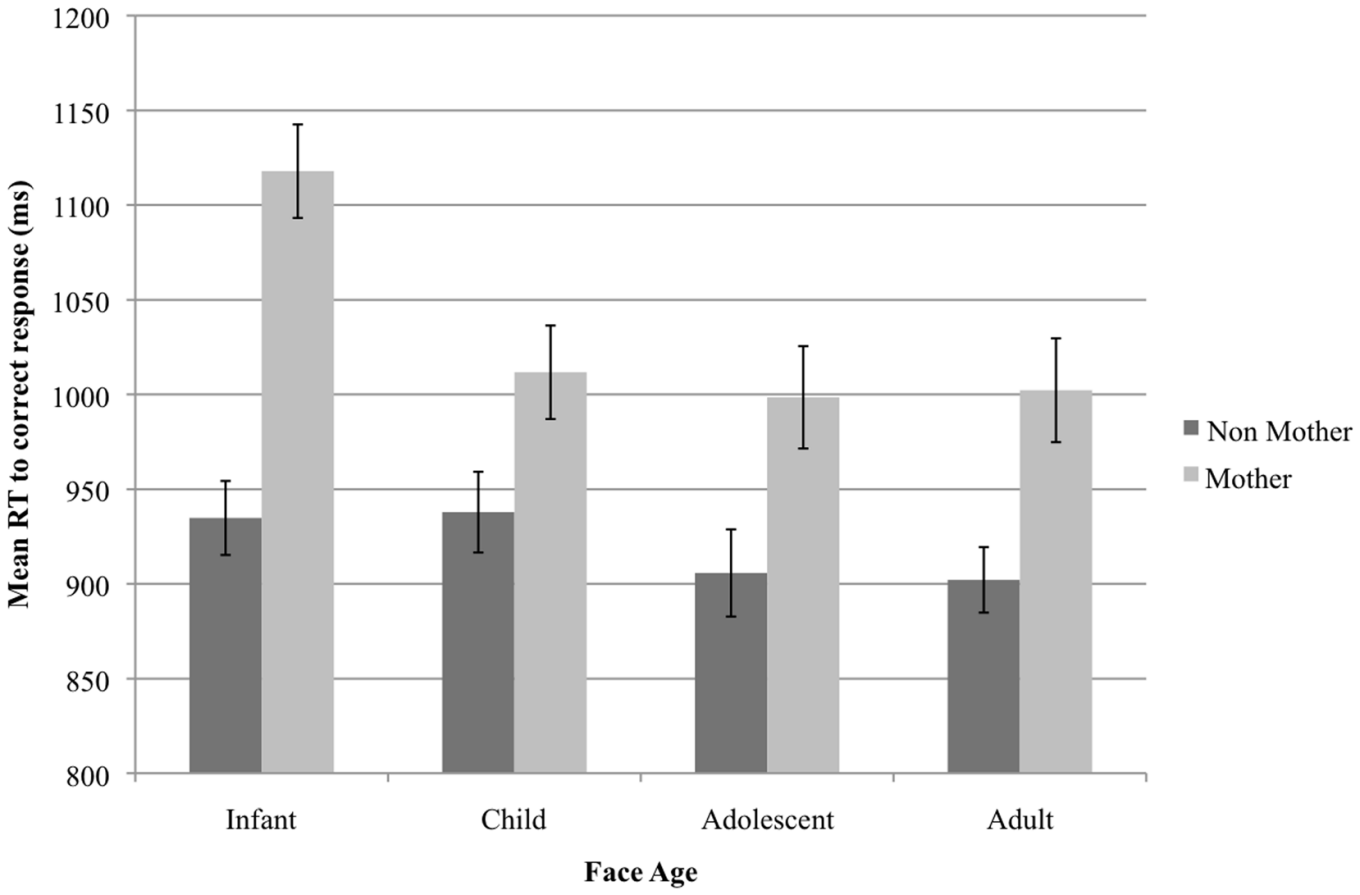
Mean RT for non-mothers and mothers as a function of face age. Error bars represent 95% confidence intervals.

There was a main effect of parent status (*F*(1, 82) = 12.98, *p*<.001, *η_p_^2^* = .14), such that mothers had longer RTs to correct responses overall compared to non-mothers (mean difference = 111.719, *SE* = 31.01). There was also an interaction between face age and parent status (*F*(3, 246) = 6.01, *p*<.001, *η_p_^2^* = .07). To investigate this interaction, ANOVAs were performed separately for mothers and non-mothers on RT data. For non-mothers, it was found that RTs to correct responses were slower in infant than in adult conditions (mean difference = 32.76 ms, *p*<.05, *d* = .43). However, RTs were not slower in infant conditions as compared to adolescent (mean difference = 29.09, *p* = .54, *d* = .26) or child conditions (mean difference = 4.12, *p* = 1.0, *d* = .04).By contrast, for mothers, RTs to correct response were slower in infant face conditions compared to child (mean difference = 104.46 ms, *p*<.01, *d* = .61), adolescent (mean difference = 119.44 ms, *p*<.001, *d* = .77), and adult face conditions (mean difference = 119.10 ms, *p*<.001, *d* = .96). Therefore slowed RTs in the infant face conditions are particularly pronounced for mothers (*see* *Figure 3*). The three-way interaction between face age, search condition, and parent status was not significant (Greenhouse-Geisser corrected *F*(5.07, 415.36) = .64, *p* = .67, *η_p_^2^* = .01).

In summary, overall RTs were slowed to infant faces as compared to other aged faces (child, adolescent, and adult), particularly in the presence of a sad affect. Responses were slowest when a target infant face displayed a sad facial expression. RTs for target faces of children were also slowed, but only when they displayed sad affect. Finally, women who were parents, as compared to those without children, displayed greatest task interference when processing infant faces.

## Discussion

This study investigated attentional processing of infant, child, adolescent, and adult faces in a group of first-time mothers of infants and women without children. We found that RTs were slowed in the presence of infant faces compared to faces of adults, adolescents, and children, with greatest task interference when infants displayed sad affect. An interaction between face age and parent status indicated that mothers' task responses were particularly slowed by infant faces. However, RTs were also slowed in the presence of child faces as compared to adolescent and adult faces, but only when the target child face displayed sadness. By contrast, attentional allocation to adolescent faces were comparable to that observed for adult faces suggesting that adolescent faces do not demand preferential attention.

Our findings in relation to infant faces are in line with previous work showing that infant faces more readily engage our attention compared to adult faces [1], [21], [22], [37] and extend these findings by establishing that infant faces are also more salient than juvenile faces from other age groups. This is perhaps not surprising given the high degree of vulnerability that characterizes the infancy period, during which adult care and nurturance is critical for survival. The unique perceptual configuration of infant faces is thought to signify this vulnerability and need for care, increasing the likelihood of eliciting caretaking responses [11], [27]–[29]. However, the results from this study only provide a speculative basis to consider a possible evolutionary function of increased attentional allocation to infant faces; it will be necessary to replicate these findings in a range of samples across cultures in order to infer that such responses are universal.

Our findings, consistent with previous behavioural and neuroimaging studies [1], [38], also suggest that parenthood is associated with enhanced allocation of attention to infant faces. It is possible that biological changes associated with becoming a parent may partly account for changes in the way infant cues are processed and prioritized [38], [46]. Equally, however, it may be the case that mothers have greater cumulative experience of viewing and responding to infant cues than non-mothers which in turn lead to changes in attentional allocation. In other words, the observed differential pattern of attention allocation may simply reflect a familiarity or “expertise” with infant faces resulting from greater exposure, rather than due to any biological changes associated with parenthood.

An experience-based explanation of increased attention to infant faces in mothers would be consistent with studies of own-age bias (OAB) and other-age effects (OAE), in which faces of varying ages are presented in upright and inverted positions to participants during recognition tasks. The rationale of these studies is that those faces with which individuals are most familiar will be processed configurally, such that speed and accuracy in recognition tasks will be impaired when these faces are inverted, whereas inversion will not impair recognition performance for those faces with which individuals are less familiar [24], [47]. For example, it has been found that people with more experience of infant faces, such as maternity ward nurses and mothers who have grown up with younger siblings, show inversion effects for both infant and adult faces, whereas those with less experience of viewing infant faces show inversion effects for adult faces only [48], [49]. This suggests that expertise with infant faces may affect perceptual processing of these faces, and thus may account the present study's finding that mothers of infants demonstrate greater attentional processing of infant faces. Future studies of mothers and non-mothers with varying degrees of childcare experience, as well as longitudinal investigations in women before, during and after pregnancy are needed to shed further light on the role of expertise and maternal biological changes in relation to attentional allocation to infant faces.

It should also be noted that mothers had slower responses overall than non-mothers. One possibility is that the slower RTs seen in mothers as compared to non-mothers across all face ages reflects an increase in attention to social and emotional stimuli in general in mothers. Future studies are required to investigate this hypothesis further by including non-social comparison stimuli in order to establish whether mothers are slower than non-mothers to faces only, or whether they have slower reaction times in general.

Given previous evidence from Proverbio and colleagues (2011) that women show larger neural responses to child faces than to adult faces (although not as large as responses to infant faces), we had expected that faces of children would receive enhanced attentional allocation as compared to adolescent or adult faces, but to a lesser extent than that observed for infant faces. Contrary to our initial prediction, we observed no differences in responses towards faces of children displaying neutral affect compared to adolescents or adults. This suggests a steep decline in facial saliency between infancy and early childhood that may parallel the diminishing strength of the “baby schema” as the child ages [11], [31], [42]. Furthermore, although the need for adult care is required beyond infancy, as children grow they are able to communicate their needs verbally, without relying entirely on vocal, facial and bodily cues. One possibility is that facial cues become less important as children become more verbal. However, child faces displaying sadness were associated with a significant increase in task interference compared to sad adolescent or adult faces. In other words, developmental age and affective state appear to interact to preferentially engage attention. It is possible that sad affect in children of this age (as in infancy) signals enhanced vulnerability and need for care, compared to expressions of sad affect in older individuals. Although beyond infancy children are able to do a number of things independently and can verbalise their needs, they still require continued parental input to meet their needs, to assist in regulating emotional distress, and to support cognitive development.

We did not find that adolescent faces received enhanced attentional allocation as compared to adult faces, suggesting the facial cues of adolescents, at least in this sample, are processed in an equivalent manner to adult faces. While adolescence is typically characterised as a time of growing independence and autonomy during which peers begin to play a more salient role, these young people typically also require a degree of continued parental attention and input [50]–[54]. The shift in attentional allocation by adults does not, however, necessarily imply that adults disregard adolescent needs. Rather, adult support may be elicited in different ways, particularly in the context of advances in adolescent verbal and mentalization abilities. Future studies are required to explore how parenting responses change across children of different ages.

We observed a generic impact of sad facial affect in slowing responses across all ages, compared to responses to neutral faces. This is in line with the broader attention literature, which suggests that facial affect, particularly negative affect, preferentially engages our attention [7], [15], [16]. Unlike anger, which signals potential threat and vulnerability to self, sadness is a powerful social cue that can signal reduced dominance in either males or females [55]. However, in both infants and children where developmental age is likely to indicate reduced status within a hierarchy, sadness may be more relevant in cuing vulnerability than relative dominance. Consistent with this hypothesis, we observed that sadness in infants represents a particularly powerful cue, enhancing attentional allocation to these stimuli. In the absence of verbal communication, enhanced saliency of infant affect is likely to be adaptive in eliciting protection and nurturance from adults. However, it should be noted that the current study did not include a positive affect condition. It is therefore not possible to infer that negative affect specifically (rather than affect in general) drives attentional allocation to infant faces. It should also be noted that the attentional capture effect for sad non-target faces was not as strong as the effect seen for sad target faces. It is possible that the specific demands of the current task may have attenuated the influence of non-target ‘distractors’ on attention. For example, a previous study required participants to search for target faces based on the gender discrimination (‘search for the male face’), which is not practical with infant stimuli [14]. In the current study, participants were requested to search for the infant or adult face with a pre-specified eye colour, which focuses attention to the eye area of non-target images, whereas gender discrimination requires holistic processing of the whole face. One consequence of this directed attention may be to reduce holistic face processing and therefore potentially minimize processing of the facial affect in non-target distractors [56].

There are some limitations to this study. As we only recruited first-time mothers of infants, it is possible that mothers show altered processing of child cues congruent with their own-age offspring. It may be that mothers with older children show enhanced attentional allocation to child faces beyond infancy, due to their experience of caring for older children or motivation to respond to children who resemble their own child in age. The experience of caring for older children may enhance attentional allocation towards and necessary and appropriate responding to these children, perhaps due to a heightened understanding of the needs of such children. In other words, while there is an inherent understanding within society that infants are vulnerable, it might be that an understanding of the vulnerabilities of older aged children is enhanced by the experience of caring for them and learning about their needs. Alternatively, as already discussed, facial cues may be less important for children who can verbalise their needs. Future studies with parents who have older children (for example, pre-school, pre-adolescent, and adolescent children) are needed to investigate the role of differential familiarity or experience with children of particular ages. Another limitation is that this study focused only on women, therefore it is not possible to make inferences about gender differences, nor to generalise these findings to men (fathers and non-fathers). It would be of particular interest to investigate paternal face-processing of infants, a surprising omission in the literature to date.

In summary, these findings suggest that age and affect are relevant in shaping attentional responses to infant and child faces. Infant faces are extremely salient, and receive enhanced attention as compared other-aged faces, particularly when they are expressing sadness. Unsurprisingly, women who are parents of infants show the greatest task interference when processing infant faces. However, child faces also preferentially engage attentional allocation relative to adolescent and adult faces, but *only* when they expressed sadness. By contrast, adolescent faces, whether or not they display sadness, were processed similarly to adult faces. These findings are an important replication and extension of previous studies, demonstrating that while infant faces are highly salient and demand increased attentional allocation even compared to other-aged child faces, child faces when displaying negative affect also elicit preferential attentional processing. It is possible that negative affect in children may signal enhanced vulnerability, compared to expressions of negative affect in adolescents who are less dependent on adult care. On the basis of these findings, it appears that the attentional system may be calibrated to respond to relevant “vulnerability cues” (notably young developmental age and emotional state), perhaps reflecting an adaptive mechanism promoting care-giving responses from adults.

## Supporting Information

Supplementary Information S1 **Stimuli ratings.** Age, valence, emotional arousal and vulnerability ratings for stimuli (N = 14).
(DOCX)

Supplementary Information S2 **Reaction time data.**
(XLSX)
