# Supplementary material for: I Can't Take My Eyes Off of You: Attentional Allocation to Infant, Child, Adolescent and Adult Faces in Mothers and Non-Mothers
Source: PLoS One. 2014 Oct 29;9(10):e109362. doi: 10.1371/journal.pone.0109362 (PMC4212970; doi:10.1371/journal.pone.0109362)
Supplement: Supplementary Information S1 — Stimuli ratings. Age, valence, emotional arousal and vulnerability ratings for stimuli (N = 14). (DOCX) [file pone.0109362.s001.docx]

S1. Stimuli ratings

Age, valence, emotional arousal and vulnerability ratings for stimuli (N=14).

Participants were asked to estimate the age of the different stimuli to ensure that they perceived the faces as belonging to the relevant age category. A one-way ANOVA revealed that face age ratings differed according to the face age category (*F*(2,52)=2284.39, *p*<.001). Post-hoc comparisons with Bonferroni correction applied revealed that infant faces were rated as younger (*M*=.81 years, *SE*=.05) than pre-adolescent child faces (*M*=5.23 years, *SE*=.19, *p*<.001), adolescent faces (*M*=13.98 years, *SE*=.17 *p*<.001), and adult faces (*M*=43.18 years, *SE*=.76, *p*<.001). Pre-adolescent faces were rated as younger than adolescent faces (*p*<.001) and adult faces (*p*<.001). Finally, adolescent faces were rated as younger than adult faces (*p*<.001).

Participants were also asked to rate all stimuli for valence on a scale of 1 (negative) to 5 (positive). A 4 (Face age: infant, pre-adolescent, adolescent, adult) x 2 (Emotion: neutral or sad) repeated-measures ANOVA was conducted on the valence ratings. There was no main effect of face age (*F*(3,39)=.29, *p*=.88). There was a main effect of emotion (*F*(1,13)=770.73, *p*<.001, *η_p_^2^*=.98), with sad faces (*M*=1.14, *SE*=.04) rated as more negative than neutral faces (*M*=2.99, *SE*=.05). There was no face age by emotion interaction (*F*(3,39)=1.07, *p*=.37).

Participants were also asked to rate the stimuli for emotional arousal. A 4 (Face age: infant, pre-adolescent, adolescent, adult) x 2 (Emotion: neutral or sad) repeated-measures ANOVA was conducted on the emotional arousal ratings. There was a main effect of face age (*F*(3,39)=45.94, *p*<.001, *η_p_^2^*=.78). Post-hoc comparisons with Bonferroni correction applied revealed that infant faces were rated as more emotionally arousing than adult faces (mean difference=1.33, *SE*=.14, *p<.*001), adolescent faces (mean difference=1.22, *SE*=.14, *p<.*001), and pre-adolescent faces (mean difference=.47, *SE*=.15, *p*<.05). Pre-adolescent faces were rated as more emotionally arousing than adult (mean difference=.87, *SE*=.14, *p*<.001), and adolescent faces (mean difference=.76, *S*E=.12, p*<*.001). There were no differences in emotional arousal ratings between adolescent faces and adult faces (mean difference=.11, *SE*=.11, *p*=1.0). There was a main effect of emotion (*F*(1,13)=67.44, p<.001, *η_p_^2^*=.84), with sad (*M*=3.49, *SE*=.05) rated as more emotionally arousing than neutral (*M=*2.38, *SE*=.14). There was not a significant stimulus by emotion interaction (*F*(3,39)=2.51, *p=.*07).

Finally, participants were also asked to rate the stimuli for perceived vulnerability on scales of 1 (low) to 5 (high). A 4 (Face age: infant, pre-adolescent, adolescent, adult) x 2 (Emotion: neutral or sad) repeated-measures ANOVA was conducted on the vulnerability ratings. There was a main effect of face age (*F*(3,39)=78.75, *p*<.001, *η_p_^2^*=.86). Post-hoc comparisons with Bonferroni correction applied revealed that infant faces were rated as more vulnerable than adult faces (mean difference=1.81, *SE*=.18, *p<.*001), adolescent faces (mean difference=1.46, *SE*=.13, *p<.*001), and pre-adolescent faces (mean difference=.51, *SE*=.10, p<.01). Pre-adolescent faces were rated as more vulnerable than adult (mean difference=.13, *SE*=.16, p<.001), and adolescent faces (mean difference=.96, *S*E=.13, p*<*.001). Finally, adolescent faces were rated as more vulnerable that adult faces (mean difference=.36, *SE*=.08, *p*<.01). There was a main effect of emotion (*F*(1,13)=55.70, *p*<.001, *η_p_^2^*=.81), with sad faces (*M*=3.47, *SE*=.05) rated as more vulnerable than neutral faces (*M*=2.55, *SE*=.11).

There was a stimulus by face age interaction (*F*(3,39)=8.99, *p*<.001, *η_p_^2^*=.41). Post-hoc comparisons with Bonferroni correction applied revealed that for neutral conditions, infant faces were rated as more vulnerable than adult neutral faces (mean difference=2.14, *SE*=.19, *p<.*001), adolescent neutral faces (mean difference=1.84, *SE*=.17, *p<.*001), and pre-adolescent neutral faces (mean difference=.68, *SE*=.14, p<.01). Pre-adolescent neutral faces were rated as more vulnerable than adult neutral faces (mean difference=1.16, *SE*=.19, *p*<.001), and adolescent neutral faces (mean difference=1.46, *S*E=.20, p*<*.001). However, there was not a difference in vulnerability ratings between adolescent neutral faces and adult neutral faces (mean difference=.30, *SE*=.10, *p=*.07). For sad conditions, infant faces were rated as more vulnerable than adult sad faces (mean difference=1.48, *SE*=.20, *p<.*001) and adolescent sad faces (mean difference=1.07, *SE*=.13, *p<.*001). However, the difference between infant and pre-adolescent sad faces did not quite approach significance (mean difference=.34, *SE*=.11, *p*=.06). Pre-adolescent sad faces were rated as more vulnerable than adult sad faces (mean difference=1.14, *SE*=.17, *p*<.001), and adolescent sad faces (mean difference=.73, *SE*=.11, *p<*.001). Finally, adolescent sad faces were rated as more vulnerable than adult sad faces (mean difference=.41, *S*E=.11, *p<*.05). Thus, the difference in vulnerability ratings between infant and pre-adolescent faces is only significant in neutral conditions, while the difference between adult and adolescent faces is only significant in sad emotional conditions. Furthermore, although other differences between baby, child, adolescent and adult faces exist for both neutral and emotional conditions, the mean difference between the ratings for the different ages is reduced slightly for sad as compared to neutral conditions.
